# Key factors to facilitate locally driven family planning programming: a qualitative analysis of urban stakeholder perspectives in Africa and Asia

**DOI:** 10.1186/s12992-021-00717-0

**Published:** 2021-07-03

**Authors:** Lisa Mwaikambo, Sarah Brittingham, Saori Ohkubo, Ruwaida Salem, Denis Joel Sama, Fatimata Sow, Deepti Mathur, Nneoma Nonyelum Anieto

**Affiliations:** 1 The Challenge Initiative, Johns Hopkins Center for Communication Programs, Baltimore, MD USA; 2 Now with FHI 360, Durham, NC USA; 3 The Challenge Initiative, East Africa Hub, Jhpiego, Kampala, Uganda; 4 The Challenge Initiative, Francophone West Africa Hub, IntraHealth International, Dakar, Senegal; 5 The Challenge Initiative for Healthy Cities, India Hub, Population Services International, Delhi, India; 6 The Challenge Initiative, Nigeria Hub, Johns Hopkins Center for Communication Programs, Abuja, Nigeria

**Keywords:** Family planning, Local government, Local ownership, Health system strengthening, Evidenced-based interventions, Scale-up

## Abstract

**Background:**

There has been greater recognition of the importance of country ownership in global health and development. However, operationalising country ownership to ensure the scale up and sustainability of proven interventions remains elusive at best. To address this challenge, we undertook a thematic analysis of interviews collected from representatives of local governments, public health systems, and communities in poor urban areas of East Africa, Francophone West Africa, India, and Nigeria, supported by The Challenge Initiative (TCI), aiming to rapidly and sustainably scale up evidence-based reproductive health and family planning solutions.

**Methods:**

The main objective of this study was to explore critical elements needed for implementing and scaling evidence-based family planning interventions. The research team conducted thematic analysis of 96 stories collected using the Most Significant Change (MSC) technique between July 2018 and September 2019. After generating 55 unique codes, the codes were grouped into related themes, using TCI’s model as a general analytical framework.

**Results:**

Five key themes emerged: (1) strengthening local capacity and improving broader health systems, (2) shifting mindsets of government and community toward local ownership, (3) institutionalising the interventions within existing government structures, (4) improving data demand and use for better planning of health services, and (5) enhancing coordination of partners.

**Conclusion:**

While some themes feature more prominently in a particular region than others, taken together they represent what stakeholders perceive to be essential elements for scaling up locally-driven health programmes in urban areas in Africa and Asia.

## Background

Scaling up a programme for widespread adoption remains a challenge in global health, despite the existence of effective and innovative products, practices, and interventions. On average, it takes 9 years for research evidence to be implemented into practice [1]. Accelerating scale up of a hypothetical 20-year global health programme by just 1 year can reach 10% more people, resulting in a significant impact on lives saved [2].

As a result, scaling up proven global health interventions requires donors, programme implementers, and stakeholders to rethink how programmes are funded, structured, and implemented. Traditionally, global health programming has been top-down, driven by the major donors and governments of high-income countries: “It has involved interventions in the name of others though frequently without their explicit sanction, control, or even participation” [3]. Since the 2005 Paris Declaration on Aid Effectiveness, there has been greater recognition of the importance of country ownership in development. However, operationalising country ownership to ensure the scale up of proven global health interventions that lead to sustained impacts remains elusive at best.

To address this issue, The Challenge Initiative (TCI), a Bill & Melinda Gates Foundation-funded project, aims to rapidly and sustainably scale up evidence-based family planning and adolescent and youth sexual and reproductive health (AYSRH) interventions, in genuine partnership with local governments across poor urban areas through its ‘business unusual’ model. TCI’s business unusual model is guided by six principles: *(1) Demand-driven:* local governments self-select and bring their own finances and ideas for addressing their family planning issues to the table; *(2) Local ownership and system readiness:* local governments must be ready, willing, and able to address their issues by leading implementation of evidence-based interventions; *(3) Right-fitting interventions:* TCI works with local governments to ensure evidence-based interventions are adapted and responsive to the particular nuances of local conditions and context [4]; *(4) Coaching support:* TCI provides support to local governments to design, manage, and implement programmes themselves; *(5) Leveraging and activating existing structures and systems:* TCI works within existing government- and community-led systems to harmonize strategies, plans, funding, and technical assistance and avoid duplication, waste, and missed opportunities; *(6) Using near- to real-time data:* TCI stresses the importance of adaptive management techniques by using data for problem solving and better decision-making.

In September 2017, three cities of East Africa self-selected to partner with TCI to implement evidence-based family planning interventions. As of February 2021, TCI’s business unsual model and evidence-based interventions have scaled to 104 cities in East Africa (Kenya, Tanzania, and Uganda), Francophone West Africa (Benin, Burkina Faso, Côte d’Ivoire, Niger, and Senegal), India, and Nigeria. The Gates Institute for Population and Reproductive Health, housed at the Johns Hopkins Bloomberg School of Public Health, manages TCI’s implementation through four partners in four regional hubs: Jhpiego in East Africa; IntraHealth International in Francophone West Africa; Population Services International (PSI) in India; and Johns Hopkins Center for Communication Programs (CCP) in Nigeria. This study aims to investigate the key factors most frequently identified by country-based stakeholders in low- and middle-income urban settings in the implementation and scale up of evidence-based family planning and AYSRH interventions. The authors then seek to reflect upon how the findings align with guiding principles of TCI’s business unusual model and what lessons can be learned from “how” TCI effects change. This analysis disrupts the current paradigm that is represented in the scale-up literature by elevating the voices of representatives of local governments, public health systems, and community stakeholders, rather than relying on interviews with “thought leaders” or public health experts largely representing high-income countries and/or academia, to define the critical elements needed for effective implementation and scale up of proven practices [5, 6].

## Methods

TCI uses the Most Significant Change (MSC) technique as its primary qualitative data collection method as a complement to its quantitative measures in order to gain a fuller understanding of not just *what* TCI is accomplishing but *how* [7]. MSC is a participatory monitoring and evaluation technique used in the implementation science field to make sense of complex program impacts in dynamic contexts, capture differences in outcomes across sites and over time as well as different perspectives on the same outcomes, and foster adaptive management [7]. This technique is particularly relevant in examining the complexities around scaling evidence-based interventions across multiple sites in 10 countries.

At its essence, MSC consists of four basic steps: (1) collecting stories of significant change from project beneficiaries or stakeholders, (2) selecting the most significant stories by selection committees at different levels of the project to surface values about what is important, (3) feeding back selected stories with all stakeholders to promote ongoing dialogue and learning, and (4) using the stories to improve the project. TCI staff across the four regional hubs collect MSC stories on a rolling basis, and selection committees within each regional hub and at the headquarters level select the most significant stories on a quarterly basis. The ultimate goal for TCI is to use the data emerging from MSC to generate learnings that inform decision-making and enhance program performance, leading to increasingly efficient processes and effective outcomes.

For this analysis, four members of the research team, who are public health practitioners/researchers engaged with TCI, conducted a secondary analysis of all of the MSC stories collected in the first year of implementation of the method. The main objective was to sharpen our understanding of the elements that representatives of local governments, public health systems, and communities perceived as critical to the sustainable scale of modern contraception in urban settings of LMICs.

### Data collection

The research team developed a semi-structured interview guide informed by the MSC technique [7]. The instrument included three main questions with additional probing:

1. In the last month/quarter, what do you think was the most significant change that occurred as a result of TCI?
2. Why do you think this is significant?
3. What are the challenges that you’ve experienced in implementing TCI’s evidence-based approaches?

Data collectors who also served as TCI coaches interviewed stakeholders through purposive sampling after receiving written informed consent. The stakeholders interviewed represented those adapting and implementing evidence-based family planning interventions and those responsible for the oversight and financing of family planning programmes at the city, municipality, county, or state level, depending on the location’s particularities. Some family planning clients and TCI project staff were also interviewed.

For this analysis, we included MSC stories collected between July 2018 and September 2019. The specific timeframe for data collection differed by region, varying from 6 months in Francophone West Africa to 15 months in India, based on when training on the MSC method occurred. In general, this timing corresponds to the second year of implementation of TCI-supported evidence-based interventions. In total, 118 MSC stories were collected during this time period. Of these, 96 MSC stories met inclusion criteria: they included information on the situation prior to TCI’s involvement, the change as a result of TCI’s involvement, and the significance of the change. Seven stories represented interviews with multiple stakeholders at the same time.

### Data management and analysis

The interview teams were trained on how to use the Voice Record app to audio record interviews using their mobile phones. However, most relied on taking detailed notes of the interviews using the semi-structured interview guide. Interview transcripts were translated into English when another language was originally used (e.g., Hindi or French).

For this analysis, the research team first organised the transcripts in a spreadsheet to track the stories by interviewer, country/region, interviewee, job title and sex of interviewee, and whether the selection committees selected the story and their rationale. The data from this spreadsheet provided the total number of stories collected during the time frame of interest and the diversity of stakeholders who were interviewed.

After examining those basic attributes, each of the first four authors then coded six transcripts manually to become familiar with the content and develop an initial set of codes, which involved a line-by-line analysis of the transcripts and coding phrases and sections of the text to summarise the data, electing to use process codes; process codes employ gerunds (−ing words) to draw out observable and conceptual action in the data [8]. This form of coding seemed particularly relevant in this context because the intention was to extract the actions and consequences of programme planning, implementation, and scale up. The research team generated the codes inductively—that is, the codes were created and iterated based on the data rather than predefining the codes a priori—and maintained a codebook of all the codes, along with the definitions. From the small sample of test stories, the research team identified initial codes that were then iterated throughout the analysis of the 96 stories. Two of the authors coded all of the stories in Atlas.ti software (version 8.4.4). To achieve intercoder reliability, the two authors each coded 20% of the stories separately and reviewed each other’s work to come to consensus before dividing the rest of the stories between themselves.

**Fig. 1:**
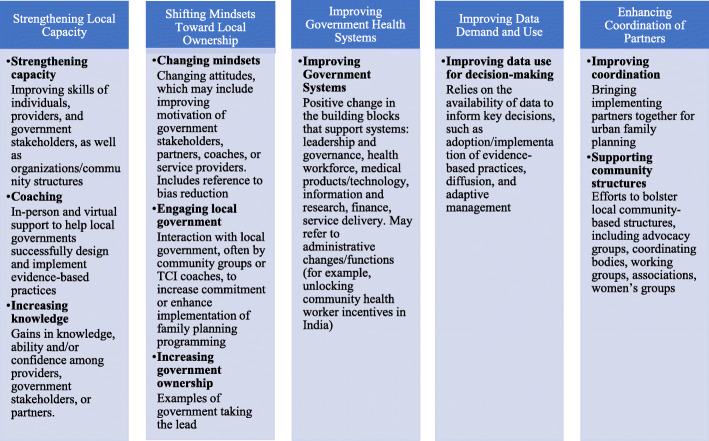
Themes, Corresponding Codes, and Code Definitions

The final analysis included 55 unique codes. Following the procedure outlined by Braun & Clarke 2006 [9] for thematic analysis, the research team analysed the codes and considered how they could be combined into overarching themes, using the six principles of TCI’s business unusual model, described earlier, as a general analytical framework. Some codes formed main themes, such as strengthening capacity and improving data use, while others were deemed irrelevant to the purpose of the study, such as background-related codes, and were excluded from further analysis. The authors iteratively refined the themes to ensure the data within each theme were meaningful and coherent while simultaneously ensuring clear and distinct differences between the themes, ultimately grouping the codes related to the aims of the study into five themes (see Fig. 1).

## Results

Of the 96 stories that were included in the analysis, nearly half (49%) were from the Nigeria hub, while East Africa and India each contributed about one-quarter of the stories, and Francophone West Africa contributed four stories (Table 1). The disproportionate collection of stories was attributable to the timing of training and delays in rolling out the MSC method due to staff turnover in Francophone West Africa. Interviewees represented a range of roles in the health system, from government stakeholders (27%) and service providers (20%) to TCI managers (12%) and community leaders (9%). Twelve percent of the stories were from family planning clients. The interviewees were relatively evenly split between women and men, with women making up 53% of interviewees. Some stories were compiled through group interviews and thus included more than one interviewee.

**Table 1:** Distribution of MSC Stories by Characteristic (*N* = 96)

Regional Hub (where stories originated)	No. (%)
Nigeria	47 (49)
East Africa	24 (25)
India	21 (22)
Francophone West Africa	4 (4)
**Role of interviewee in the health system**	
Government official	26 (27)
Service provider	19 (20)
Health client	12 (12)
TCI state or city manager	12 (12)
Community leader	9 (9)
Community health workers (CHWs)	7 (7)
Health educator/social mobilizer^a^	5 (5)
Implementing partner	3 (3)
HMIS/M&E officer	3 (3)
Other facility staff	2 (2)
**Sex of interviewee**	
Female	51 (53)
Male	38 (40)
Multiple storytellers	7 (7)

*Abbreviations*: *HMIS* health management information system, *M&E* monitoring and evaluation

^a^ Note: Health educators are government employees, but they are grouped with non-governmental social mobilizers since both health educators and social mobilizers provide information/counselling to clients, but not services

From this analysis, five key themes emerged that facilitate implementation and lead to positive changes in scale up and sustainability of family planning programmes, according to TCI’s stakeholders: (1) strengthening local capacity, (2) shifting mindsets toward local ownership, (3) improving government health systems, (4) improving data demand and use, and (5) enhancing coordination of partners. In general, the codes applied to the MSC stories in this analysis appeared across the regional hubs, but some codes featured more prominently in certain regions. We explore each of these themes and highlight notable regional variations in the sections below.

### Strengthening local capacity

The most frequently mentioned themes in the MSC stories related to capacity strengthening, with about one-third of all stories highlighting various elements linked to capacity strengthening. This trend was more prominent in India, where many of its stories described changes in local capacity. Specifically, those stories pointed to the value of embedding and aligning evidence-based interventions and tools for implementing them within existing city structures and processes to facilitate their adoption and to help government staff, especially at the facility level, perform their jobs better. For example, a service provider in Uttar Pradesh, India, explained:

This [data reporting tool] made it possible to review the health center’s family planning data during monthly government meetings … and take corrective actions including arranging human resources and family planning supplies. Having accurate data in a simple format that was being reviewed regularly encouraged better performance and motivated the facility staff and community health workers to give their best.

Many stories described various capacity strengthening approaches such as coaching, mentoring, and training opportunities that enabled stakeholders to implement, manage, and monitor evidence-based family planning interventions. Thematic analysis elucidated the characteristics of TCI’s coaching approach that they value most. For example, a government official in Bauchi state, Nigeria, referenced consistent access to coaching support:

TCI … is different from other projects [in] that the [TCI] office is embedded inside the agency. In fact, they are like staff of the agency, and so collaborating with the staff of the agency in order to provide technical support needed is within the same environment.

Furthermore, interviewees noted the outcomes of coaching support related to broader health systems functions, such as leadership, management, and coordination. A government official from Dar-es-Salaam, Tanzania, explicitly linked skill building in management, budgeting, and monitoring and evaluation to improved government leadership and service to the community:

We, the government staff, are now involved in planning, budgeting, monitoring, documenting, and evaluation... Right now, planning is done at both facility and municipal levels. Through TCI, there are so many improvements and our activity and budget plans are smart. Most of [the] challenges we used to face have been minimized. The local government knows the community needs and we now have a sense of ownership and we aim at sustaining it.

### Shifting mindsets toward local ownership

Another frequently occurring theme across the regions was changing mindsets about the important role of local governments and communities in leading family planning programmes. Altogether, about one-third of the stories covered mindset changes, and the stories from East Africa and Nigeria illustrated such outcomes more prominently than the two other regions. For local governments, the mindset shift was linked to increased political and financial commitments. At the community level, the analysis revealed how community stakeholders recognized the essential role they could play in improving the health of their communities. In both cases, the mindset changes were noted as a foundational step to motivating action among local government players and community members to improve access to and use of family planning services.

#### Local government players

Local government stakeholders often described the demand-driven approach that requires political leadership as well as financial resources for implementation as a novel one that they were unaccustomed to—one that they embraced and that prompted them to take action. For example, a health educator in Rivers state, Nigeria, described how empowering this mindset change was:

… we are seeing an NGO coming to say that “you drive the process and we follow.” … We are seeing ourselves in the driver’s seat and if we don’t do it, no one else will! It calls for ownership and participation at the state level, LGA [local government area] level, and community level. That is a very great change.

In Uganda, a service provider celebrated the mindset shift and movement toward increased ownership and accountability among city government, commenting:

Nowadays, our political leaders are interested in knowing how much of the geography money [local government budget] we are spending in family planning. … Before TCI came on board, these leaders were not asking such accountability-related questions.

Several stories described how witnessing the impact of demonstration interventions motivated them and reaffirmed the stakeholders’ commitment to implementing the evidence-based interventions. For example, a service provider of Uttar Pradesh, India, shared the significance of a demonstration of the evidence-based approach, “dedicated day for family planning services,” in which trained staff, equipment, supplies, and commodities were made available on a pre-announced day and time at an urban primary health center, had on them:

Family planning was the last thing on anyone’s mind at our urban primary health center. However, after observing and participating in the special drive [for family planning services] in 2018 facilitated by TCI, I saw people coming in for family planning services. From that day onwards, we are regularly conducting dedicated service day without the support of the TCI team.

These mindset changes also spur local stakeholders’ involvement in better understanding and responding to community needs. For example, in Plateau state, Nigeria, a government official shared how one community issue was resolved:

When the deputy governor’s wife and governor’s wife were commissioning the facilities, we learned that they [the facilities] didn’t have water. … We dug a borehole and put a tank in place to pipe the water. I was able to do this with money that the state already had available [in the existing government budget]. We didn’t know that water was an issue before TCI. The TCI model gives us an opportunity to enter to see what some of the problems are.

#### Community members

Many stories, in particular those from Nigeria, described the community as a key contributor to and implementer of the evidence-based interventions. This support or engagement with community structures has not only improved the family planning knowledge of community members but also changed their negative attitudes and perceptions of services and empowered them to take action to further help improve service delivery in their communities. For example, an implementing partner of an NGO visited Bauchi state, Nigeria, to learn from its experience implementing the clinic makeover approach and then used that learning to replicate the approach across Gombe state, a non-TCI supported state in Nigeria. A key lesson learnt that the partner took away from the experience was how the community actively took part in and owned the process for implementing the approach:

The work continued to the next day, which is Sunday … with a renewed commitment from the community representatives and the staff including the artisans themselves. In fact, you can see clearly from their faces because they have that impression of owning the process, it is their facility, it is their families who are the direct beneficiaries.

Similarly, a community leader in Bauchi state, Nigeria, described how the community’s enthusiasm has led to financial contributions to the health facility:

The community is very happy. … Before the renovation, the community donated drugs to the facility worth over N350,000. Now that the facility has been renovated, the community is working on bringing a doctor that will be resident in the facility here and treat cases.

Some stories described how the evidence-based interventions targeted key influencers, such as traditional and religious leaders and community members who served as community health workers (CHWs), to become family planning champions. A religious leader in Plateau state, Nigeria, explained that he was carrying out a door-to-door campaign among his network to share the importance of family planning and that he was sharing what he learned from TCI with other religious leaders, which sparked a broader mindset change among the Muslim clerics in his area:

There were misconceptions on the part of the Islamic clerics, they normally interpret child birth spacing as controlling population but after our interaction with them using the Islamic Perspective and Sermon notes on child birth spacing [from TCI], a lot of them have now understood that spacing in between births for the health and well-being of the mother and child is also promoted in the Holy Quran.

In several stories, CHWs from India shared their lack of confidence and discomfort in counselling on family planning during household visits. After coaches accompanied CHWs on their household visits and demonstrated how to do effective counselling, the CHWs reported that their images improved within their communities as women began to seek them out for family planning information and services. A CHW in Odisha, explained:

Since I had never done this before, I thought that the situation is going to be very embarrassing in the field and I may lose my good connection. … These [coaching sessions] were extremely useful. Now I hold these conversations independently, and rather enjoy doing so because I find that women are making their own decision about contraception.

These mindset changes in the community extended to youth as well. For example, in Benin, a government official highlighted the role of youth in implementation of AYSRH programmes:

The youth in general and young leaders are more and more responsible; as you see them taking part in activities, you can see that they are willing to do their best.

### Improving government health systems

Improving health care delivery by the public sector was another frequently captured theme. Stakeholders shared various examples of how the evidence-based interventions had been institutionalised, diffused, or scaled across the public health system. About half of the stories in India illustrated specific examples related to this theme. For instance, the local government, with coaching support, mapped and defined slum catchment areas to better define service needs and allocate resources. All government programmes, including family planning, immunisation, and maternal and newborn health, started using the slum maps to plan urban health interventions. According to a government official from Madhya Pradesh:

Defining the catchment area of each facility with ward and slum population details is helping all government health programmes including immunisation, FP, and MNH. This has resulted in improving FP and MNH service uptake and better reporting from each facility on all HMIS indicators, including FP and MNH indicators. Overall, the mapping and listing process has resulted in the strengthening of the urban health system.

Additionally, a number of MSC stories from India highlighted how advocacy efforts to streamline government systems to process timely release of incentive payment to CHWs has resulted in increased motivation among the workers. A CHW from Madhya Pradesh explained the significance of this change:

Earlier, we were not aware of our incentives or responsibilities. … Once TCI intervened, our meetings were formalised with our health center staff every Saturday. Now, we are regularly being updated about our programmes and incentives. … We are well-versed about the health worker diary [register] and the processes of voucher filling and submission, which we did not know earlier.

A government official also shared his appreciation of this change in the system:

This streamlined process of submission of vouchers has reduced the load on the medical officer in-charge and the entire system of processing of payment has been simplified. The timely release of payments of incentives has motivated the CHWs to do more work and has also helped in reducing the attrition rate of CHWs.

Several MSC stories from Nigeria described how the improved facility infrastructure from clinic makeovers supported delivery of not only family planning services but also primary health care services overall. A stakeholder from Plateau state, Nigeria, explained that the government recognized this and supported replication of the clinic makeover approach in other non-TCI supported health centers.

Analysis of MSC stories also revealed a more effective use of existing public health sector staff. For example, a service provider in Ziguinchor, Senegal, highlighted the impact of universal referral, an evidence-based intervention that the government was coached on how to implement, which involves maternal and newborn care staff screening their clients for family planning services:

Through TCI tools, a client may now be identified as needing family planning services. She is systematically given the referral sheet for necessary care and when she comes back, she is identified as having received FP services. This strategy did not exist in the past.

### Improving data demand and use

Many stories, especially the ones from India and East Africa, presented a case about improving data for decision-making. These fell into three categories: (1) the use of data as part of advocacy efforts to motivate and inspire local governments to prioritise family planning interventions; (2) strengthened capacity of local stakeholders to collect, analyse, and use data; and (3) how this capacity strengthening in data use ultimately led to a culture of data demand by both local government stakeholders and implementers at the facility level.

#### Data-informed advocacy

MSC stories described a number of approaches to fostering local ownership and investment and how those approaches were critical for implementation and scale. One approach involved direct advocacy to local governments to add a budget line item for family planning and AYSRH—and to release those funds. These advocacy efforts resulted in local government allocation and release of funds for family planning programmes in general and for AYSRH programmes specifically. In Nigeria, a TCI state manager explained:

The release of the funds is significant as it indicates government investment in the AYSRH programme, which is critical for sustainability of AYSRH programmes …

In addition, several stories described how data was used to demonstrate success of the evidence-based approaches, which then spurred systems-level changes. For example, in Uttar Pradesh, India, several stories described demonstrations of a dedicated day for family planning services in 25 sites over a two-month period. Upon reviewing the data demonstrating primary health centers’ capacity to successfully provide quality family planning services, the local government deemed the dedicated family planning day a worthy investment and scaled the approach throughout all urban primary health centers. One government official noted:

When we find out that something [is] good and workable [and] produces results quickly, we take it into the system—which is what we have done in case of the dedicated day for family planning services. This is now part of [the] center’s charter and [is] going to sustain forever. The system works, not individuals. So, when something is introduced or added into the system, no one needs to worry about its sustainability.

#### Data-improved capacity

Many of the stories referred to the significance of the coaching support provided to public sector counterparts in a number of areas, a key area being capacity to collect, interpret, and use data. For example, a monitoring and evaluation officer of Bauchi state, Nigeria, shared:

My capacity has now been built not only on how to log into the HMIS platform but how to download data, analyse, and compare it to see the differences and improvements. Not only that, my capacity has been strengthened that I collate monthly data from health facilities and conduct data quality assessment and share it with implementing partners. It wasn't like this before the coming of TCI. I feel very confident now.

Similarly, a health educator in Uganda reported improved capacity among all community outreach workers in her district:

… The Iganga District Health Team noticed a huge improvement in the capacity [of the community outreach workers] to compile and submit their reports, including those of family planning. Many of the health facilities have now incorporated VHT [volunteer health team] data into the HMIS monthly health facility report and reporting is on time. VHTs are now in position to cross check in the HMIS to ensure their data is incorporated in the health facility report.

#### A culture of data for decision-making

Several stories highlighted the use of both project-generated reporting tools and a government-owned data platform, HMIS, as ways to promote a culture of data use for decision-making. These efforts improved data accuracy and timeliness of health information data, leading to greater government ownership of the evidence-based interventions. For example, a health information focal person in Uganda shared how the integration of data from CHWs into facility registers, which was reported into the data platform, has helped target services more effectively:

Nowadays, health workers are more responsible and health facilities own their data. They ensure completeness of their FP [family planning] data reports so that their efforts are realised. Community health workers have learnt to demand for their data to be displayed at the health facilities. We now discuss our FP data regularly and the service providers are able to identify gaps and strategies to improve performance. We are able to take FP services where they are most needed.

A data assistant in Uganda noted how more accurate facility-level data was used to better target services to the population, leading to improved access to and quality of services:

Most of the facilities are now prioritising using data for decision-making, which is evidenced by graphs and charts drawn in some FP units supported by TCI. The division has observed a huge improvement in terms of FP service delivery access and quality. Through outreaches [a TCI evidence-based intervention], FP services are taken closer to the urban poor who may not afford transport to the facilities.

### Enhancing coordination of partners

The stakeholders’ stories, particularly from East Africa and Nigeria, illustrated how enhanced coordination leads to more agile, impactful programming. For example, a government official in Bauchi state, Nigeria, emphasised how she now feels empowered to coordinate the various implementing partners:

TCI provided a platform for us to see a reason for all partners to come together under one umbrella so that we could move to make things happen better and quicker with less stress but greater impact. … Now the agency knows and coordinates activities of partners. There is synergy now … every partner has a shared vision.

Several stories touched upon how the project served as a facilitator or knowledge broker in bringing together a range of stakeholders to more transparently plan and roll out implementation of evidence-based interventions, resulting in the integration of family planning into other health programmes, such as maternal, newborn, and child health, ultimately meeting women’s and families’ needs more holistically. A health educator of Rivers state, Nigeria, explained:

We were able to build family planning into our other programmes—Maternal Newborn and Child Health Week, for example … We will be having quarterly meetings at the local level to bring different stakeholders together through the help of TCI. … We are able to expand more.

Some stories illustrated how the coordination effort went beyond direct implementing partners and donors and reached out to other local government units. In Senegal, a government official described how the demand-driven approach activated coordination across three of the five municipalities comprising the region:

With TCI, our three city councils have pooled their funds to deliver FP and AYSRH services in some geographies. We will respond collectively … and this practice is unusual. All of the activities in the action plan are implemented at the same time in Bignona, Oussouye, and Ziguinchor.

Other stories pointed to the significance of enhanced coordination between the public health system and the private sector for improved family planning service delivery as a result of capacity strengthening. A service provider in Uganda said:

There is better coordination with private health facilities and we have [an] improved referral system. So, a client who does not want to come to the public health facility will still get the family planning service at the private health facilities. Before this, we were only concentrating on the public facilities. We have strengthened the capacity of the midwives providing family planning in the private sector.

This coordination also extended to improving linkages between the community and service delivery system by activating community structures. For example, strengthening women’s groups was an evidence-based practice in India that aimed to strengthen community accountability and facilitate access to family planning services among the urban poor. A government official conducted a visit to a women’s group and noted his excitement at seeing the mother’s group successfully activated:

I was enthralled to hear this amazing true story of these women champions. If these women groups were to work as a true connection between the community aspirations and health service delivery, then the day will not be far when every citizen of the country will get quality health care services provided for them by the government.

## Discussion

This analysis of nearly 100 MSC interviews elevates five key factors for ensuring locally driven family planning programmes from the perspective of local government officials, service providers, community leaders, and other health systems stakeholders from 10 countries with diverse socio-political contexts and health systems of varying maturity levels. First, *capacity strengthening* of local officials to implement evidence-based interventions as well as broader health systems functions through coaching creates a positive feedback loop, whereby cities are exposed to the tangible benefits of their efforts. The majority of MSC interviews reported on how TCI’s coaching support has led to individual and organizational capacity strengthening. Alongside this capacity, *mindsets* of both local government officials and of community members shift toward local ownership, empowering and motivating them to contribute more of their own resources to expand access to and use of health services. The demand-driven nature of TCI’s partnership with cities is reinforced and strengthened through coaching and changes in mindsets toward local ownership. Third, institutionalisation of evidence-based interventions within existing government structures and a focus on *improving government health systems* leads to diffusion of the interventions beyond the geographic areas that receive the initial investment. Leveraging and activating existing structures and systems, instead of creating parallel structures, not only strengthens local ownership but also strengthens capacity within the system. This is evidenced by TCI’s commitment to using the government-owned HMIS for monitoring and evaluating the impact of the evidence-based interventions. In addition, engendering a *culture for data demand and use* helps improve the accuracy and timeliness of data, leading to improved planning and targeting of family planning services to populations in need. Finally, improved *partner coordination* further highlights the importance of leveraging existing systems and structures, which leads to more agile, impactful programming. For example, in Nigeria, this is achieved through state-wide integrated family planning workplans developed by the state government with support from TCI to bring together all implementing partners to transparently share their individual project objectives and plans. In addition, in most poor urban cities in LMICs, it is critical that the public and private sectors work in partnership to meet the health needs of the ever-growing urban population. Although private sector partnerships were not frequently reported in our collection of stories, this is an area that TCI is actively working on with local governments and expect to see as an emerging theme in future MSC stories.

These five themes align with all six of TCI’s business unusual guiding principles. However, the demand-driven and right-fitting principles did not come out as frequently as the other principles in the MSC stories. This may be for two reasons. First, both of these principles are built into the process of engagement with TCI in which local governments decide whether to complete an expression of interest to join the TCI partnership and select which evidence-based interventions meet the needs of their specific context. As a result, the interviewees may not view these aspects of the model as part of the implementation approach but pre-implementation processes. Second, a number of the principles are interrelated. For example, demand-driven and local ownership go hand-in-hand. However, interviewees reported more frequently about the significance of the mindset shifts that they experienced toward local ownership in leading and managing their family planning programmes and implementing the evidence-based interventions than the demand-driven aspect of the model in which they commit their own financial resources to implementation. The analysis also revealed that the stakeholders view coaching local governments to align and adapt evidence-based interventions within existing government systems and structures as a significant factor, which is related to the process of right-fitting or adaptation itself.

One important aspect that emerged in our analysis that could be emphasized more in TCI’s guiding principles is *community ownership* in addition to local government ownership. Community ownership and mobilisation have been identified in a recent systematic review as crucial facilitators of intervention sustainability [5]. The current global challenges of pandemics and outbreaks also signal that local government and community ownership is required in scaling safety measures for preventing further spread of deadly viruses. There is no one shared definition or construct related to community ownership. However, it has been presented as on the pathway to effective and sustainable health interventions [10]. Communities ensure government accountability and extend the limited resources of government. Interviewees emphasised community resources as an important input when strategizing to maximise contributions and impact. Community members also served as decision-makers and implementers of the evidence-based interventions, and community ownership was celebrated alongside local government ownership in several interviews. This finding is supported in the literature related to barriers to scaling up, which include lack of engagement of local implementers and of the adopting community [11]. Subsequent research should explore whether or not perceptions of community ownership change over time, especially as engagement with TCI gradually ends.

Our results add to the body of the literature in the scale-up and sustainability fields by providing perspectives from local stakeholders themselves, rather than from public health “experts” or “thought leaders,” on important facilitating factors. These stakeholders highlighted coaching to strengthen capacity, alignment of interventions, tools, and approaches with the existing system, the use of data for decision-making, and coordination of partners as significant elements needed to facilitate local ownership and implementation of evidence-based interventions, with the ultimate goal of scale up and sustainability. These themes are applicable to the implementation of evidence-based interventions beyond family planning and are also supported by existing literature that has identified key facilitating factors for scale-up to include strong leadership and governance, active engagement of a range of implementers and of the community, and simple interventions proven to be effective and tailored to the local context with recognisable benefit to the local population [12–15]. These are also necessary factors to support the *sustainability* of evidence-based interventions and public health programmes beyond the initial funding period. In the sustainability literature, these factors include programme design elements such as stakeholder involvement and working with existing resources; organisational characteristics such as favourable organisational culture, strong leadership, and sound infrastructure; and environmental features such as community engagement and ownership [16–18].

We believe the cornerstone of successful implementation and scale efforts is coaching of local health systems actors to strengthen their capacity to design, implement, manage, and continually monitor and adapt their own health programmes to changing conditions. Shifting mindsets and fostering local ownership starts with building and cultivating leadership, which Brownson et al. (2018) noted as the most important aspects of capacity. TCI aims to strengthen local government leadership at the systems, organisational, and individual levels by systematically embedding coaching within existing local structures. Initially, our coaching approach focused primarily on strengthening capacity to scale up specific evidence-based interventions. We quickly learned that technical know-how is necessary but not sufficient to ensure sustained scale and impact of quality family planning and AYSRH programmes. Our coaching support evolved to include strengthening leadership skills, programme management, coordination, planning, budgeting, and use of data to inform decisions—skills that will help ensure sustainability of implementation beyond TCI’s support. Because our coaching approach was designed to be flexible to meet the needs of local health systems, the specific details on how coaching is implemented vary between countries and regions. A study is currently being carried out to document the “how” of our coaching approach and to assess the specific outcomes that are associated with coaching.

### Limitations

There are some limitations with this analysis that should be noted. Since the data collectors come into regular contact with the interviewees through coaching sessions, there may be a courtesy bias in which the interviewees report mainly on positive changes and/or the data collectors select to interview individuals who have had positive experiences. In addition, the five countries in which TCI is implemented in Francophone West Africa are underrepresented in this analysis due to staffing turnover and, therefore, challenges in providing coaching support to local governments and limited ability to collect interviews. Finally, this analysis represents the early experience of implementing evidence-based family planning and AYSRH interventions by TCI stakeholders (within the first 2 years of implementation). At a later point when TCI begins to withdraw its coaching support, the findings may change.

## Conclusion

When local leaders are in the driver’s seat of their health programmes, evidence-based interventions are able to reach more people and have greater impact, especially when communities themselves are empowered and involved in planning and implementation. The analysis of nearly 100 MSC interviews with local implementers and stakeholders of four LMIC regions highlights five key themes as important factors in the implementation and scale-up of locally driven health programmes: (1) strengthening local capacity to implement evidence-based interventions and improve broader health systems, (2) shifting mindsets of government and community toward prioritising family planning programmes and local ownership, (3) institutionalising the interventions within existing government structures, (4) improving data demand and use for better planning and targeting of health services, and (5) enhancing coordination of partners. While some themes feature more prominently in a particular region than others, taken together they represent what stakeholders perceive to be essential elements for scaling up locally-driven health programmes in urban areas of LMICs.

This analysis is timely as external funding from traditional donors is decreasing at the same time that their expectations to make the most of their funding is rising. Governments in LMICs are being called upon to increase domestic spending and co-finance health programmes while new mechanisms, such as the Global Financing Facility, are being introduced, driving a shift from grants toward mixed funding [19]. In this environment, the findings from this analysis can influence how other programmes and innovations are designed for implementation and scale.

## Data Availability

Johns Hopkins University Data Archive https://doi.org/10.7281/T1/SWNGEP
